# Factors affecting the production of sugarcane yield and sucrose accumulation: suggested potential biological solutions

**DOI:** 10.3389/fpls.2024.1374228

**Published:** 2024-05-13

**Authors:** Faisal Mehdi, Zhengying Cao, Shuzhen Zhang, Yimei Gan, Wenwei Cai, Lishun Peng, Yuanli Wu, Wenzhi Wang, Benpeng Yang

**Affiliations:** ^1^ National Key Laboratory for Tropical Crop Breeding, Institute of Tropical Bioscience and Biotechnology, Chinese Academy of Tropical Agricultural Sciences, Haikou, China; ^2^ Sanya Research Institute, Chinese Academy of Tropical Agricultural Sciences, Sanya, China

**Keywords:** environmental stresses, biocontrol agents, resistance genes, sugarcane, sucrose accumulation, tolerant varieties, yield

## Abstract

Environmental stresses are the main constraints on agricultural productivity and food security worldwide. This issue is worsened by abrupt and severe changes in global climate. The formation of sugarcane yield and the accumulation of sucrose are significantly influenced by biotic and abiotic stresses. Understanding the biochemical, physiological, and environmental phenomena associated with these stresses is essential to increase crop production. This review explores the effect of environmental factors on sucrose content and sugarcane yield and highlights the negative effects of insufficient water supply, temperature fluctuations, insect pests, and diseases. This article also explains the mechanism of reactive oxygen species (ROS), the role of different metabolites under environmental stresses, and highlights the function of environmental stress-related resistance genes in sugarcane. This review further discusses sugarcane crop improvement approaches, with a focus on endophytic mechanism and consortium endophyte application in sugarcane plants. Endophytes are vital in plant defense; they produce bioactive molecules that act as biocontrol agents to enhance plant immune systems and modify environmental responses through interaction with plants. This review provides an overview of internal mechanisms to enhance sugarcane plant growth and environmental resistance and offers new ideas for improving sugarcane plant fitness and crop productivity.

## Introduction

Environmental stress can negatively affect plant growth and productivity, particularly sugarcane crop. Understanding plant biochemical and physiological responses to environmental factors, stress mechanisms, and potential crop tolerance strategies is crucial to mitigate such effects. Sugarcane is a major C4 crop primarily grown in tropical and sub-tropical regions and is essential for sugar and bioenergy production. Climate-related factors, such as temperature, light, water, precipitation, and extreme weather are crucial for global sugarcane production (Zhao and Li, 2015). However, climate change has led to drought, heat stress, insect pests, and diseases, which limit crop productivity. Pests and diseases, including approximately 100 fungi, 10 bacteria, 10 viruses, and 50 nematodes, contribute to declining crop yield worldwide (Rott et al, 2000). Sugarcane yield losses are primarily caused by diseases, such as rust (20%), smut (75%), ratoon stunting disease (RSD) (40%), and mosaic virus infection (40%), which lead to 37% global agricultural production loss, and 13% are attributed to insects (Butt et al., 2016). Many countries have reported diseases and insect pests affecting sugarcane. For instance, approximately 1300 insect pests attack sugarcane crops globally, with Pakistan having 61 species responsible for such attacks (Qamar et al., 2021). More than 360 insect pest species have been reported on sugarcane, and they cause 15%–20% yield loss in China (Shang et al., 2024). Abiotic factors, such as heat stress, in plants can significantly constrain productivity, thereby decreasing the production of different agronomical species from 2.5% to 10% (Hatfield et al., 2011). High temperatures can significantly affect photosynthesis, respiration, water balance, and membrane stability in leaves and lead to reduced crop yield (Cao et al., 2022). Sugarcane growth requires temperatures between 8°C and 34°C for CO_2_ absorption during winter, while chilled temperatures (below 8°C) hinder photosynthesis, causing stunted leaf growth. High temperatures in tropical and subtropical regions negatively affect sugarcane germination, resulting in low plant populations, increased short nods and stem fibers, and decreased sucrose content (Bonnett et al., 2006; Rasheed et al., 2011). Drought influences sugarcane production by reducing root water availability and causing water loss through transpiration. It also reduces agricultural production by affecting photosynthesis, growth, and nutrient–water relationships. Plant responses vary by species and environmental factors, and the major yield-reducing factors are limited soil moisture, reduced radiation absorption, and decreased harvest index. In Brazil, 9.1% of sugarcane yield decreased due to drought stress, causing up to 60% losses (Gentile et al., 2015). Environmental stressors cause protein or enzyme denaturation, membrane damage, elevated reactive oxygen species (ROS), cellular disruption, and DNA damage, which then reduce crop yield and affect sugarcane crop growth. Heat stress can lead to drought and disease infections (Wahid et al., 2007). Microorganisms within and outside plants’ tissues include bacteria, fungi, archaea, algae, and protists. Throughout their long evolutionary history, these species have evolved intricate networks, which ultimately culminate in symbiosis instead of leaving them as separate organisms. This crosstalk among microbiomes and plants has a positive effect on plant existence, fitness, and ecological function (Sasse et al., 2018). This crosstalk is the most common example of a symbiotic relationship between arbuscular mycorrhizal fungi and rhizobia in legume plants. The main feature of a symbiotic relationship is the early development of certain cells, tissues, and organs for communication and feeding exchanges between microbes and plants (Zipfel and Oldroyd, 2017). However, endophytic relationships may potentially be crucial for plant fitness (Khaled et al., 2018). Plants with endophytes and poisonous alkaloids exhibit strong resilience to environmental pressures (De Oliveira Chagas et al., 2017). Research revealed that endophytic relationships are crucial for plant immune system, disease resistance, nutrient acquisition, and resilience to abiotic stressors (Khan et al., 2015). Many studies have been conducted on endophytes in other plants; however, the mechanisms through which endophytes assemble in sugarcane plant tissues and manage to survive without causing any symptoms remain unclear. Furthermore, we elucidate approaches by which these endophytes enhance sugarcane plant productivity and survival. This review examines the effect of environmental factors on sucrose content and cane yield formation and highlights the potential of endophytes as biocontrol agents.

## Biotic stresses affect sugarcane

### Sugarcane fungal diseases

Research identifies 39 disease species, including 22 fungal, 3 bacterial, 1 mycoplasma, and 2 viral diseases, in China (Jinju et al., 2013). A recent work reported more than 120 sugarcane diseases worldwide, with over 60 found in China, which can cause yield losses of up to 20% (Huang et al., 2018). The main sugarcane fungal diseases are sugarcane smut, red rot, Pokkah boeng pineapple disease, etc. Sugarcane smut, a fungus found in countries, such as Pakistan, Brazil, and China, significantly affects the global sugar industry because it reduces commercial crop production. Yield losses associated with this fungus range from 17% to 22% in South Africa and from 10% to 15% in Hawaii and Florida. The fungus reduces cane stems, resulting in sugar loss (Deng et al., 2018). The most dangerous disease that affects the growth of sugarcane is smut disease, which is caused by *Sporisorium scitamineum.* Smut, a disease causing 100% infestation in sugarcane fields, affects 84 planted crops and 80 ratoon crops, leading to a 9%–75% loss in production and a 3%–7% decrease in sugar recovery (Malik, 2018; Ali et al., 2022; Ali et al., 2023). According to research conducted on Australian commercial sugarcane Q157, a substantial relationship exists between the severity of smut disease and yield, with an average loss of 26%–62% (Magarey et al., 2010). Sugarcane smut is a severe disease that significantly affects China’s sugarcane sector because it causes low sucrose content and yield (Cai et al., 2021). Four vulnerable varieties, namely, Chauntang 61-408, Guitang 11, Guitang 12, F134, and Co419, have been eliminated (Huang et al., 2018). Other susceptible varieties, including ROC22 and Mingtang, also suffer from the disease. In this regard, sugarcane smut resistance cultivars (YT93-159, ZT-2, YC05-179, and YZ05-51) are recommended (Wu et al., 2022). Nitrogen fertilizer significantly affects sugarcane productivity, that is, the yield declines by 10% in resistant varieties and by 50% in vulnerable varieties; under harsh conditions, the susceptibility to smut disease increases. Significant scientific progress has been achieved in the fields of biological breeding and smut disease resistance mechanisms. Joshi and Goswami (2024) claimed that *Trichoderma* may combat sugarcane smut disease by generating substances that improved the pathogen-reducing antagonistic effect against *Sporisorium scitamineum*, leading to 22.8% to 66.9% reduction in the incidence rate of smut disease decreased from, respectively. Meanwhile, it increased agricultural production from 25.2% to 49.8%. In this study, *Trichoderma* is a potential plant growth fungus that may be utilized for effective treatment of several sugarcane diseases, including smut. A previous study on *Pseudomonas aeruginosa* B18 isolated from sugarcane revealed that the strain contains genes related to biological control mechanisms, colonization, and biofilm formation, which are linked with secondary metabolite metabolism. Hence, *Pseudomonas aeruginosa B18* has an important role in plant growth and biological control mechanisms in sugarcane (Singh et al., 2021a). Furthermore, many studies have reported that biocontrol agents, such as *Bacillus*, *Pseudomonas fluorescens*, and *Trichoderma* spp., can inhibit the growth of *Sporisorium scitamineum* (Tegene et al., 2021; Wang et al., 2021; Rees et al., 2022; Vijitrpanth et al., 2023). Most bacteria, including the denitrification-active *actinomycete Marmoricola*, *Iamia*, and *Reyranella*, are helpful to smut resistance (Duan et al., 2023). Endophytic fungi, such as *Ramichloridium*, *Alternaria*, *Sarocladium*, *Epicoccum*, and *Exophiala*, have been reported as antagonistic to sugarcane smut (Chen et al., 2019). Current findings suggest that endophytic bacteria, such as *Pseudomonas aeruginosa* and *Cyphellophora*, are effective for controlling smut disease.

Red rot, also known as “sugarcane cancer,” is a severe disease that significantly affects sugarcane stubble and yield in many countries and causes economic losses of about 1/3 of cane culms. In India, the yield loss associated with this disease reached 100% (Viswanathan, 2021). The disease affected 100% of the land in Pakistan’s sugarcane-producing region, resulting in 83% cane yield loss and a 31%–75% sugar recovery rate (Malik, 2018). Sugarcane yield and sucrose content declined by 5%–50% due to the disease affecting 100% of the land. A study on two sugarcane varieties, S2003-US-127 and CPF-250, showed resistance against red rot and reported high yield and sugar recovery rates (Malik, 2018; Ahmad et al., 2022). *Bacillus velezensis*, a biocontrol agent, is widely used in agriculture because of its efficacy and environmental safety (Sun et al., 2022). Endophyte (*Bacillus velezensis YC89*) extracted from sugarcane leaf can suppress the growth of red rot pathogen by 78% (Xie et al., 2023). Hence, *Bacillus velezensis* can promote plant growth and biocontrol.

*Fusarium moniliformae*, which was first identified by Sheldon (1904), is confirmed as the cause of pokkah boeng in Asian sugarcane-producing regions. The disease severity varies from 5% to 90% (Vishwakarma et al., 2013) and can cause 40.8%–64.5% sugar content reductions, thereby affecting high sugar-yielding cultivars (Raghvendra et al., 2021). In Pakistan, pokkah boeng affected 90% of cane and led to a 17%–84% cane yield loss and a 7%–10% sugar decline (Malik, 2018). In China, this disease has been reported in various provinces, with Guangxi experiencing an outbreak with a 52.4% infestation rate and a 14% decline in sugarcane yield (Huang and Li, 2016; Shan et al., 2021). The China’s sugarcane sector faces increasing danger from this disease because it spreads to sensitive cultivars, such as Yuetan 57-423, ROC1, ROC10, ROC16, ROC22, ROC25, and Yuetang54-176, with infestation rates ranging from 30% to 80%. Sugarcane infestation rates can reach 81.1% to 100%, with sugar content declining up to 4%. This disease is a death warning for sugarcane production and spreads due to improper cultivation practices, high temperatures, and humidity (Shang et al., 2024). Chinese researchers have developed elite cultivars to combat this disease but have not evaluated their resistance. Biocontrol agents, such as *Aspergillus flavus* (56.92%), *Aspergillus niger* (55.38%), *Trichoderma viride* (81.50%), *Trichoderma hamatum* (50.76%), and *Trichoderma harzianum* (70.76%), have shown inhibition rates, respectively (Srivastava et al., 2019).

Pineapple disease is a destructive sugarcane disease that harms the root system and produces an odor similar to that of fully ripe pineapples. The term “pineapple disease” originates from the aroma of ethyl acetate produced by the causative agent, *Thielaviopsis paradoxa* (Talukder et al., 2007). This pathogen contaminates sugarcane setts and seeds and leads to irregular germination and destruction of sugarcane stands. It can cause a 50% reduction in sprouting and reduce the yield by more than 42% (Chapola et al., 2014). This pathogen thrives under conditions with long-term humidity and low temperatures. It can be controlled by soaking sugarcane in lime water or carbendazim-wettable powder for 24 hours.

### Sugarcane bacterial diseases

Ratoon stunting disease (RSD)is a global disease caused by bacterium *Leifsonia xyli or Xylite* and significantly affects sugarcane yield. Infestation can reduce yield by 24% in L99-226 and 32% in L99-3233 crops, causing more than 50% yield losses in sugarcane (Grisham et al., 2009). Certain varieties of sugarcane, such as Guitang11, Guitang94-119, Yuetang93-159, and Yuetang00-236, have the highest RSD incidence rates, suggesting the need to focus on healthy seedling development (Li et al., 2014). Further research is needed to determine the extent of resistance and the potential for breeding resistant sugarcane cultivars. The disease reduces cane yield by 37% for first-planted crops and 29% for ratoon crops. It also affects the source-to-sink relationship in sugarcane and macronutrient absorption due to the pathogen attached to the root tip (Shang-dong et al., 2020; Garcia et al., 2021). Hot water treatment is the most cost-effective method for managing RSD, with 2 hours treatment using 50°C water as suitable. Endophytes can regulate and enhance cane yield and brix ton per hector (Carneiro et al., 2021). A study on sugarcane cultivars revealed the high diversity of resistant cultivars, suggesting that they could be potential biocontrol agents for xylem pathogen colonization and potentially affect plant health (Gao et al., 2022).

Leaf scale disease, one of the most prominent bacterial infections of sugarcane, is caused by *Xanthomonas albilineans* and has a major negative economic effect on the global sugarcane sector. Leaf scale reduces juice quality, especially in the ratoon crop, leading to high cane losses measured in tons per hectare (Gutierrez et al., 2018). *Xanthomonas albilineans* invades the vascular system of sugarcane leaves, stems, and parenchyma cells (Mensi et al., 2014). The disease has been identified in Guangxi Province, China, in new breed varieties, namely, Guitang46 (44.6%) and Guitang06-2081 (50.1%) (Zhang et al., 2017). These findings suggest that both varieties are susceptible to leaf scale. This disease also reduces sugarcane output and sucrose content. Plants that are severely infected by leaf scale disease show several symptoms, including wilting, aberrant side branch growth, and side shoots with white-striped leaves (Li et al., 2018). A recent work reported that leaf scale infected two chewing cane sugarcane clones (Guangdong Huangpi and Taoshang Guozhe) in Zhejiang province (Duan et al., 2021).

*Acidovorax avenae* causes top rot and red stripes in sugarcane plants (Hernandez-Juarez et al., 2021), resulting in striped and red leaves. These symptoms can occur independently or concurrently, depending on environmental factors, such as temperature and humidity. Red-stripe (RS) disease has increased in frequency and severity over the past decade and caused significant economic losses. Elevated temperatures encourage the transmission and spread of the microbe to new locations in cane fields (Yonzone and Devi, 2018). The frequency of the disease is influenced by novel production methods and vulnerable cultivars (Grisham and Johnson, 2014). The disease reduces sugarcane stem availability, yield, and juice quality (Fontana et al., 2016). RS affects 40% of sugarcane-growing areas and causes reductions of 6% sugarcane yield, 16.13%–48.39% sucrose content, and 10% sugar recovery rate (Malik, 2018). According to Yonzone and Devi (2018), this bacterium affects the sugarcane growing area of the variety CoJ85 by 56%–54.33% in India. In China, this disease was reported in six sugarcane-producing provinces, including Yunnan, Hainan, Guizhou, Guangxi, Guangdong, and Fujian. The findings indicate that RS is widely distributed throughout China, indicating the need for control measures to mitigate its adverse effect on sugarcane yield.

### Sugarcane viral diseases

**Figure 1:**
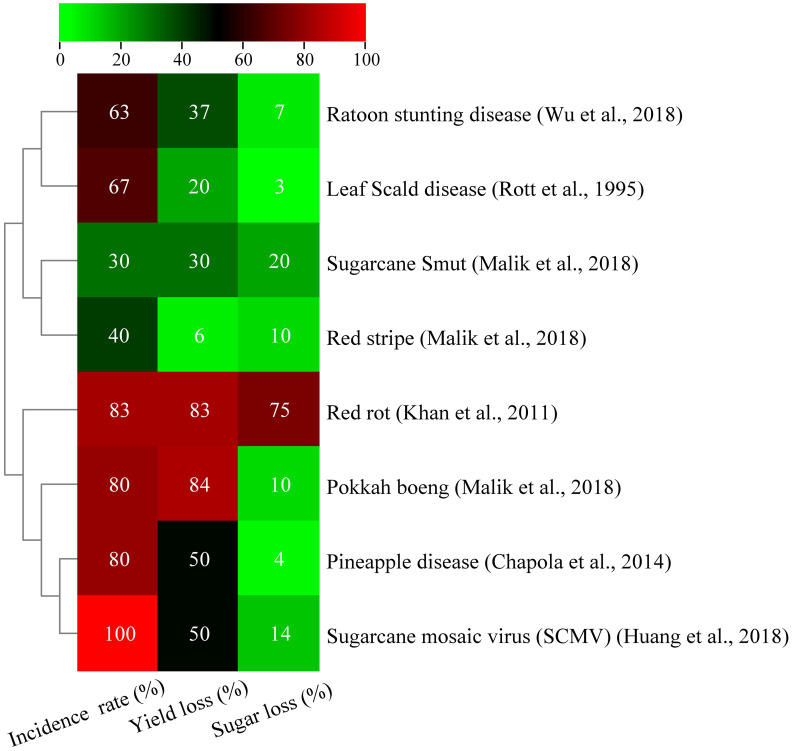
Effect of various bacterial, fungal, and viral diseases on sucrose content and yield of sugarcane (Rott et al., 1995; Wu et al., 2018).

Sugarcane mosaic disease, which was discovered in Java, Indonesia, in 1892, has affected major countries worldwide (Grisham, 2000), causing yield decreases of 17% to 50% in vulnerable varieties (Bagyalakshmi and Viswanathan, 2021). Sugarcane mosaic symptoms are currently linked to various diseases caused by different viruses. Mosaic viruses include sugarcane mild mosaic virus (SCMMV), sugarcane striate mosaic-associated virus (SCSMV), sorghum mosaic virus (SrMV), and sugarcane mosaic virus (SCMV) (Rott et al., 2008). The majority of viruses, including SCMMV, are transmitted through contaminated mechanical instruments, aphids, and infected cuttings, and the pink mealybug is the primary mode of transmission (Gonçalves et al., 2020). These viruses have been reported in various countries, including the US, India, China, Brazil, and Argentina (Nithya et al., 2021; Sun et al., 2021). Sugarcane mosaic disease in Brazil caused significant yield losses and threatened industry collapse in 1920–1930. Despite controlling damage with resistant cultivars and healthy sett planting, more than 50% yield losses, reduced juice quality, and sett germination led to several cultivar discontinuations (Gonçalves et al., 2012; Pimenta et al., 2023). In Pakistan, sugarcane field yield loss is 8%–40%, sugar recovery rate is 2% (Afghan et al., 2022). In India, SCMV incidence ranges from 14% to 90% in commercial varieties (Krishna et al., 2023). Sugarcane mosaic disease can considerably reduce sugarcane germination, photosynthetic efficiency, yield, and quality. Infected plants produce fewer stalks, thereby affecting the harvest value. Pandemic outbreaks have also caused significant economic losses in the industry, leading to financial difficulties and even bankruptcies. Developing virus-resistant sugarcane varieties and rational planting are crucial to prevent and control the disease. This disease has led to the eradication of several varieties of sugarcane (Krishna et al., 2023). Advances in genetic engineering and molecular marker-assisted breeding have significantly accelerated the development of tolerant cultivars, thereby improving the breeding process of multi-resistant crops. Furthermore, genetic engineering can produce sugarcane cultivars that are resistant to this disease. RNA interference technology has developed disease-resistant transgenic sugarcane plants to improve resistance to mosaic disease and increase yield; however, regulations have prevented their application in field production. Thus, biocontrol agents can be used to control this disease. All fungal, bacterial, and viral diseases have severe effects on sugarcane yield and sugar content (**Figure 1**).

### Insect pests

**Figure 2:**
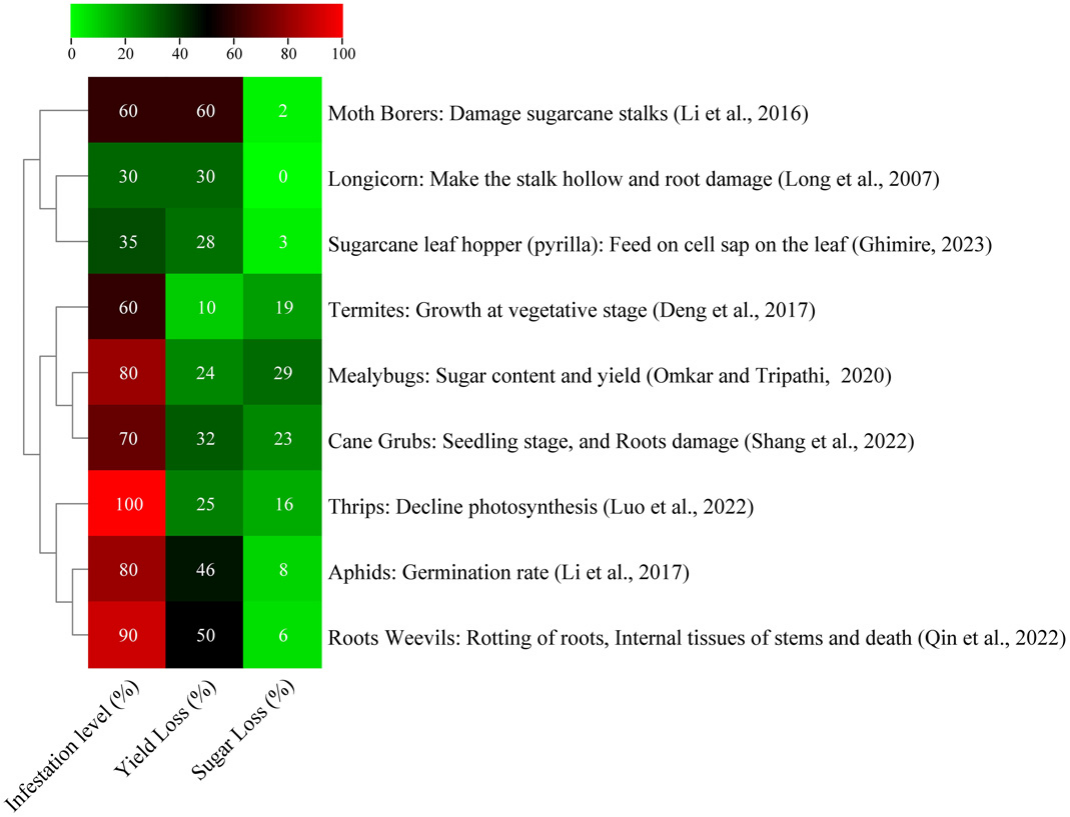
Effect of different insect pests on sugarcane yield and sugar content (Deng et al., 2017; Li et al., 2016; Li et al., 2017; Omkar and Tripathi, 2020; Qin et al., 2022; Ghimire, 2023).

Diseases and pests cause 37% of agricultural productivity losses worldwide, and 13% of which is due to insects (Butt et al., 2016). Sugarcane borers, also known as *Diatraea saccharalis*, are the main insect pest. In Brazil, larvae in 25.77% and 19.01% of internodes resulted in projected losses of 8.80% and 19.80% in sugar production per 1% of bored internodes (Rossato et al., 2013). A study in France found that highly susceptible varieties SP71-8210 and R579 experienced decreased sugar yield and production loss due to sugarcane stem borer damage (Bi Pene et al., 2018). Pakistan, among the top five sugarcane-producing countries, faces numerous borer species that can cause significant yield loss. These species include stem borers, top borers, root borers, and gujarat borers. The most harmful insects are sugarcane leaf hoper and white fly, which can reduce crop output by 25% and 15%–25%, respectively. These insects pose significant threats to the country’s sugarcane industry (Qamar et al., 2021). Common sugarcane leaf pests in China include *Chilo infuscatellus*, *Chilo sacchariphagus*, *Tetramoera schistaceana*, *Scirpophaga excerptalis*, *Sesamia inferens*, *and Chilo auricilius*, with three to seven generations annually influenced by temperature, light, and rainfall (Xu et al., 2013). The China’s sugarcane sector faces challenges due to high reproduction rates, generational overlap, and extended damage periods. Borer infestation has led to a 40%–60% decline in sugarcane production, with yield decreasing by 10.20 tons/ha in Guanxi and Chongzuo (Tan et al., 2011). In Guangdong province, the yield loss reached 10.21% and infestation rates 26.7%–96.7% were observed (Long et al., 2013). These pests significantly harm sugarcane yield and reduce sugar content, causing complete crop loss in some plots (Shang et al., 2024). **Figure 2** shows a list of certain pests that seriously harm sugarcane yield and reduce sugar content.

Thrips are historically a key sap feeder and can cause adverse effects on sugarcane at all growth stages. In China, thrips are found in almost all sugarcane-producing regions, including Yunnan, Guangdong, and Guangxi. Weather parameters significantly affect damage intensity, and damages caused by thrips are severe during moisture stress conditions, such as drought, waterlogging, and slow sugarcane growth. According to Zhang et al. (2008), the infestation level of thrips can be 100%, causing a 10%–25% decrease in sugarcane production. Frequent drought and waterlogging in certain Guangxi sugarcane areas have significantly affected crop’s susceptibility to severe thrip damage (Yin et al., 2015). Additionally, a susceptible cultivar (YT93-159) lost 40.58% of its yield, which was threefold greater than the loss of a resistant cultivar (YZ05-51) (Shang et al., 2024). A previous study on different sugarcane varieties revealed that the variety Yurui 06-189 attracted more thrips per plant compared with Mintang 01-77. Moreover, a ratoon crop of variety ROC22 was found to be susceptible to thrips (Yin et al., 2015). This finding suggests that the number of thrips varies among different varieties, and ratoon crops are more susceptible than planted crops. Hence, planting timing or date is also important to avoid thrip infestations.

*Dorysthenes granulosus* larvae feed on sugarcane roots, causing hollowing out of the stalk and severe damages to plant yield and sucrose content. This pest was found in different provinces of China, including Guangdong, Hainan, Nanning, Zhanjiang, and Guangxi. According to Zeng and Huang (1981), the typical infestation level of this pest is 5%–10%, but it can range from 30% to 40% during severe infestations. At the start of this century, *Dorysthenes granulosus* outbreaks occurred in Guangxi, resulting in destruction of millions of hectares in 2005 (Yu et al., 2007). In some areas of China, such as Beihai, Nanning, Chongzuo, and Liuzhou, the damage level is normally 15%–20%; in some areas, the infestation level reaches 40%–50%, which is considered a major threat to sugarcane. Damaged sugarcane plots had eight larvae per stump, leading to heavy cane yield losses of 15–30 tons/ha (Long and Wei, 2007). The use of prevention and control measures, such as traps (e.g., light and deep pits) and insecticides, has led to a gradual decline in the *Dorysthenes granulosus* population and infested areas (Yu et al., 2020).

The black sugarcane beetle (*Alissonotum impressicolle Arrow*) was observed in clay–loamy soil in two main sugarcane-producing provinces, namely, Yunnan and Guanxi sugarcane fields. The mature beetle that feeds at the bottom of seedling sugarcane causes the development of dead hearts in the seedlings. In severe situations, about 70% of sugarcane seedlings are infested. Numerous cane grub species can cause damages to sugarcane roots, which drastically reduces the yield (10%–20%) loss and sugar content (Shang et al., 2022). Guangxi has 20 counties, which are affected by pests, with Baise and Hechi experiencing the worst infestations and Yichou having a seedling mortality rate exceeding 40%. Ratoon populations were more severely affected than sugarcane plantations (Shang et al., 2014). Another grub called *Exolontha castanea* feeds on sugarcane, which results in lodging, drying, and losses of 32% and 23.3% in sugarcane production (Shang et al., 2016). Grubs feeding on the roots of seedlings cause gaps in the field at the establishment stage. At the grand growth and maturity stages, grub infestation leads to lodging and drying, thereby considerably affecting sucrose content and sugarcane yield (An and Guan, 2010). The overall insect pest damage is summarized in **Figure 2**.

## Abiotic stresses affect sugarcane

### Drought stress

**Figure 3:**
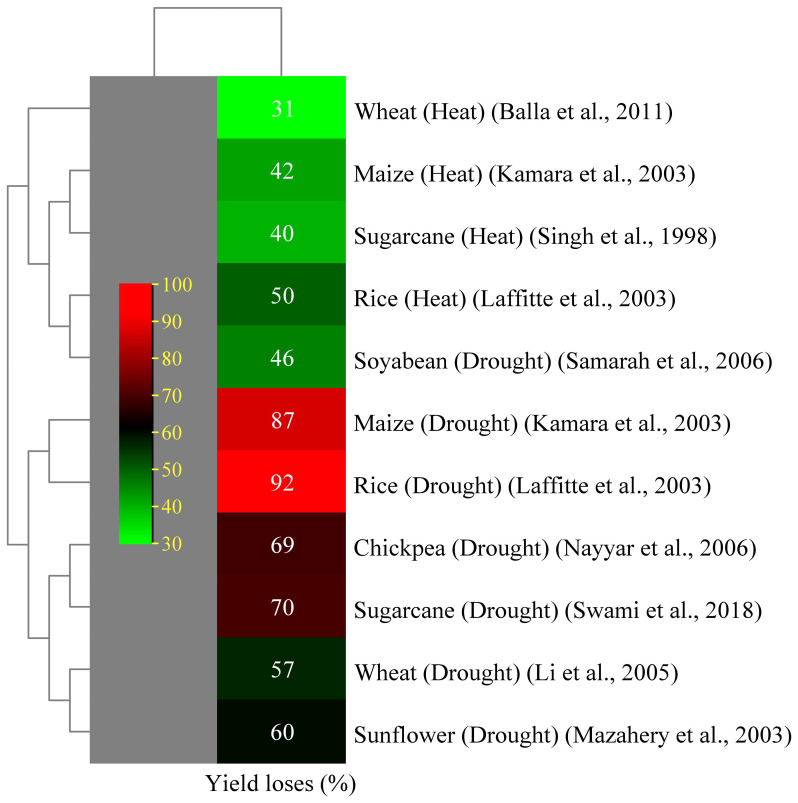
Effect of abiotic stress on yield decline of several crops (Singh and El Maayar, 1998; Kamara et al., 2003; Lafitte et al., 2003; Mazahery-Laghab et al., 2003; Li et al., 2005; Nayyar et al., 2006; Samarah et al., 2006; Balla et al., 2011).

Drought can cause partial or total production loss in plants and hinder seedling germination and development. Drought can be classified as agricultural or meteorological, which occur when soil moisture levels fall below the necessary growth and production levels (Farooq et al., 2009). Lack of water causes turgor loss, which impairs the flow of nutrients from the xylem to the surrounding cells, slows down photosynthesis, and stunts the growth of leaves (Hussain et al., 2008). Drought-tolerant cultivars have significant canopy cover, reduced temperatures and transpiration rate, and increased stomatal conductance, allowing them to grow and perform well in stressful situations (Ferreira et al., 2017). Drought-susceptible cultivars wilt, resulting in reduced total cane yield (Ogbaga et al., 2020). Research indicates that sugarcane is vulnerable to drought stress at the tillering, elongation, and grand growth stages. Drought stress considerably affects leaf growth and stem elongation. Many studies have reported that a decrease in green leaves on sugarcane stalks led to a decline in sucrose accumulation given that juvenile leaves make a sufficient contribution (Hussain et al., 2018; Hoang et al., 2019). A prolonged dry season reduces the sucrose content and juice purity because the sugarcane plant consumes its accumulated sucrose for metabolism (Begum et al., 2012). The sucrose to non-sugar ratio of sugarcane determines the quality of its juice (Xiao et al., 2017). Adequate hydration during the maturity phase boosts the sucrose production by focusing on absorbed carbon dioxide (CO_2_) on sucrose synthesis and stalk formation (Inman-Bamber, 2004). Agriculture yield is indicated by crops grown per unit area and is influenced by biological processes. Drought stress negatively affects physiological functions, with its effect varying based on stress intensity and plant growth phase (phenology). Swami et al. (2018) reported 70% yield losses in sugarcane crops, with sugar recovery losses of 15%–45% (Sanghera and Jamwal, 2019). Drought is a major factor that contributes to the decline in sugarcane yield and production worldwide (Khaled et al., 2018; Misra et al., 2020). Sugarcane cultivation faces challenges due to insufficient rainfall and extreme temperatures, thereby limiting growth and development. The first indication of damage from drought is phenotypic change, which includes decreased tiller production, discoloration, leaf shredding, and rolling (Misra et al., 2016a; Misra et al., 2016b). Plants undergo morphological changes in response to severe water stress, such as drought, either as a defense mechanism or as a result of cellular component damage (Dinh et al., 2017). Sugarcane primarily rolls its leaves in response to drought to reduce the amount of surface area accessible for light absorption and water loss (Inman-Bamber and Smith, 2005). When soils are moist, sugarcane roots expand laterally; however, when soil moisture content is low, the roots are not uniformly distributed and grow deeply. The fundamental role of lateral roots is to absorb nutrients, so root structure alterations brought on by nutrient deficits can impair yield (Hoang et al., 2019). The potential for osmotic pressure in plant tissues is directly correlated with the amount of water in the soil. Plant physiological activities are altered so they can adapt to their environment. These alterations include hormonal alterations and reductions in photosynthetic rate (Khan et al., 2015). Under drought conditions, the primary causes of decreased photosynthetic rates are as follows: (i) decrease in the relative water content of plants (Khonghintaisong et al., 2018); (ii) stomatal closure to reduce the amount of transpiration (Hoang et al., 2019); and (iii) damages caused by reactive oxidative species to the chloroplast (Li and Assmann, 2009). During drought stress conditions, biochemical and photosynthetic activity in the mesophyll and bundle sheath cells is reduced due to low water availability. This phenomenon leads to a reduction in sugarcane yield and sucrose formation because photosynthesis is the primary mechanism underlying plants’ ability to produce sugar (Sage et al., 2013). Abiotic stressors are responsible for yield reductions in several important crops (**Figure 3**).

## Effect of fertilizers on sugarcane

Agronomists emphasize the importance of controlling key nutrients, namely, micronutrients, such as iron, zinc, and boron (B), as well as macronutrients, such as nitrogen (N), phosphorus (P), potassium (K), and magnesium (Mg), to improve crop yield. The production of sugarcane, a key crop, can be enhanced by understanding the effect of long-term nitrogen fertilization. Research suggests that using 300 kg/ha of nitrogen fertilizer for the first ratoon and 150 kg/ha for the second and third ratoon crops can maximize crop yield, sucrose formation, stalk yield, and sugarcane content (Fortes et al., 2013). Nitrogen fertilizer affects the entire growth cycle of the sugarcane plant. However, different countries have different nitrogen application rates. For example, the recommended nitrogen fertilizer rates are 60–100 kg N/ha in Brazil, 160–200 kg N/ha in Australia, and 100–755 N/ha in China (Robinson et al., 2011). Brazil has higher sugarcane production despite its lower nitrogen application rate than the other countries. Sugarcane cultivation in Brazil is highly efficient because of favorable growing conditions and produces maximum yields even with low nitrogen fertilizer input compared with other sugarcane-producing countries (Bordonal et al., 2018). Optimizing fertilizer management techniques can improve crop nitrogen use efficiency and reduce environmental impacts. Inadequate fertilizers can lead to reduced biomass and sugar yields, early ripening, stunted plants, and other negative effects. Phosphorus (P) is crucial for adenosine triphosphate formation and promotes root growth and photosynthetic activity. Insufficient P levels decrease root development, and strict management of available P levels is necessary to achieve high sucrose and sugarcane yields. Potassium (K) is essential to maintain osmotic potential and store sucrose. K deficiency in sugarcane can decrease bud germination, drought resistance, and growth. Additionally, micronutrients are essential for sugarcane production, with magnesium playing a crucial role in sucrose accumulation and transportation. Silicon (Si) enhances sucrose accumulation and increases Zinc (Zn) bioavailability, thereby promoting pest and disease resistance, improving leaf quality, and reducing lodging. During the grand growth period, maintaining a balance between development and storage is crucial for nutrient supply. Boron (B), a crucial element in sugar transport, can negatively affect sucrose formation. Treatment with B improves sugarcane quality (Ferraz de Siqueira et al., 2013). Iron (Fe) converts ethylene precursors into ethylene, which is essential for cane maturity and sucrose formation. Zn is essential for plant development, sucrose synthesis, water efficiency, and chlorophyll production (Calcino et al., 2018).

### Heat stress

**Figure 4:**
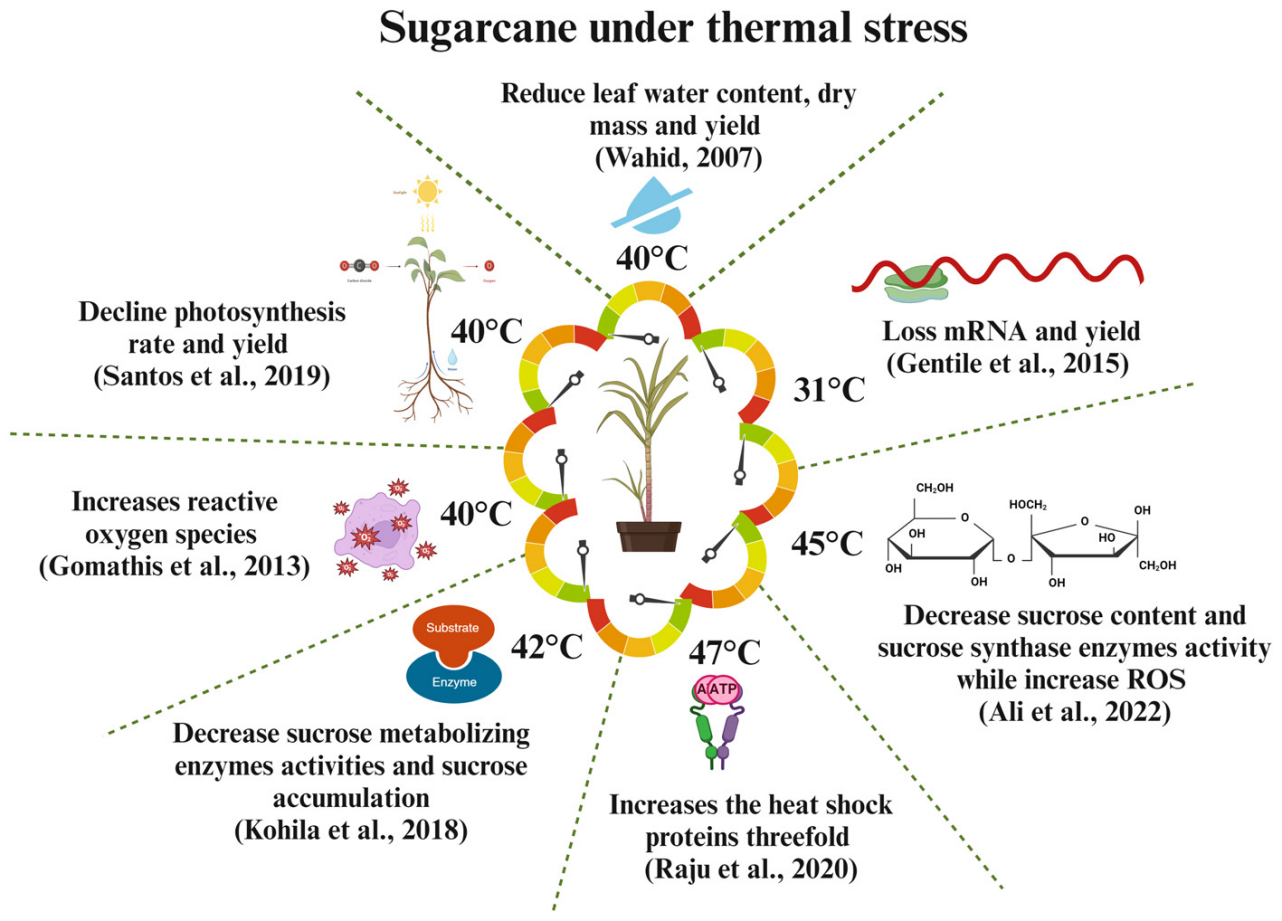
Responses of sugarcane crops to different thermal challenges. High temperatures affect the molecular, physiological, and biochemical processes of sugarcane, leading to reduced yield and sucrose production (Wahid, 2007; Gomathi et al., 2013; Raju et al., 2020).

Heat stress threatens global food security by reducing crop yield. The global food production must increase by 70% to meet the food demand of 9 billion people by 2050. Understanding heat stress mechanisms is crucial to develop thermotolerant crop varieties. Climate-related events, such as temperature, precipitation, and carbon dioxide, affect sugarcane yield and sucrose formation. Sugarcane growth is stunted at temperatures above 36°C (Wahid et al., 2007), and the ideal temperature is between 8°C and 34°C. Chilled temperatures reduce photosynthesis and leaf growth, leading to lower sugarcane yields. Additionally, an 8° slope on cane farms can further affect yield (Ram et al., 2007). Heat stress in tropical areas slows down plant growth and development, leading to increased node growth and stem fiber content, short internodes, and decreased sucrose concentration. Heat can also cause leaf senescence, inhibit growth, and reduce yield and photosynthesis rate (Bonnett et al., 2006). Heat stress can affect sugarcane fuzz (true seed) germination, potentially reducing its capacity to germinate or affecting sett; the effects change according to the degree and length of exposure to heat stress. In maize and sugarcane, heat stress dramatically reduces growth, yield, and net assimilation rate. It also causes early leaf senescence and decreases internodal length, yield, and biomass accumulation (Wahid and Close, 2007). The water status of plant is crucial in situations where temperatures fluctuate. When the supply of moisture is adequate, plants often attempt to stabilize their tissue water content regardless of temperature variations. However, if the temperature increases to a deadly level, then water will be insufficient. Even though soil has plenty of water, sugarcane exhibits a rapid loss in the amount of water in its leaf tissue when exposed to heat stress (Wahid and Close, 2007). Heat stress leads to increased water loss during the day due to high transpiration rates, thereby compromising plant physiological functions. It also decreases root quantity, mass, and development, restricting water and nutrient availability to above-ground portions. However, limited data exist on the direct effects of heat stress on crop nutrient relationships (Huang et al., 2012). Sucrose-metabolizing enzymes (Kohila and Gomathi, 2018) and nutrition metabolism enzymes, such as nitrate reductase (Klimenko et al., 2006), are significantly decreased under heat stress conditions, leading to reduced root biomass and nutrition absorption in the root. Overall, environmental challenges, such as drought and high temperatures, hamper different physiological processes that affect the nutritional cycle, absorption, and availability of plants. Fahad et al. (2017) examined the effect of heat and drought stress on crop nutrition, including essential nutrients, such as nitrogen, phosphorus, potassium, magnesium, and calcium. Sugarcane, a C4 plant, has high photosynthesis efficiency that is affected by high temperatures and water stress. This phenomenon is attributed to factors, such as reduced leaf growth, abnormal photosynthetic machinery, and leaf senescence. Drought-induced stomatal closure decreases CO_2_ availability and increases plant vulnerability to photodamage. Heat and drought stress harm the photosynthesis mechanism, causing pigment alterations and enzyme activity reduction, leading to significant plant development and yield losses (Vu et al., 2001). The above findings suggest that heat stress has negative effects on sugarcane nutrition, yield, and sucrose formation during the biosynthesis of sucrose, transportation, and accumulation into the stem. Hence, we can propose that the improvement of thermotolerant varieties can be a significant approach to enhance sugarcane yield formation and sucrose content. **Figure 4** illustrates how heat stress affects sugarcane production and sucrose formation.

### Relative humidity

The amount of sucrose content and sugarcane yield are typically unaffected by relative humidity. However, under extreme conditions, relative humidity considerably affects sugarcane productivity. Sugarcane grows quickly in environments with 80%–85% humidity and warm temperatures. Low water and moderate relative humidity are ideal throughout the ripening phase (Srivastava and Rai, 2012). Long periods of bright sunshine, optimal rain fall, and high humidity enhance rapid plant growth and increase sugarcane length to obtain high yields. The ripening stage is a crucial phase of sucrose storage and process and needs clear sunshine, warm days, dry weather, and about 51% relative humidity. About 12% of the sugarcane’s weight and 15% of its height are reached during this period (Srivastava and Rai, 2012). During the grand growth period, the risk of evapotranspirational demand is significant. Regular irrigation of the crop utilizing surface water and groundwater resources satisfies its active growth and water requirements. The cane elongation phase, which occurs during the monsoon season and lasts from July to September, is a crucial stage in plant growth. This stage is characterized by an optimal temperature of 28°C–32°C. When the temperature decreases below 20°C and the relative humidity ranges from 60% to 65%, sugarcane growth and development slow down. The rapid sucrose formation or accumulation commences from October to December. Throughout the maturation phase, the climate affects the ultimate sugar output; cold, dry weather with considerable daily temperature changes and enough soil moisture are ideal (Moore and Botha, 2013).

### Light intensity

The growth of sugarcane crops relies on photosynthesis, a process that conserves energy for plant fibers and sugar and produces four-carbon sugars (C4). The daily carbon gain from photosynthesis is influenced by latitude and cloud cover. Sunlight intensity is crucial for the grand growth stage of cane plants because it speeds up stabilizing ranges and photosynthesis rates. During the tilling formation period, a cloudy and short-day season has a significant effect on the plant. However, a warm temperature with bright sunshine (around 7 to 9 h) is favorable for tillering, culm formation, and improved growth and development (Fageria et al., 2010). Proper row-to-row and plant-to-plant spacing is necessary to provide the sugarcane crop with the right amount of sunshine, which will directly affect production. The sugarcane’s upper six leaf canopies block 70% of the sun’s energy due to reciprocal shade, thereby decreasing the rate at which leaves carry out photosynthesis. Short-growing areas benefit from close spacing for high yields, while long-growing areas need wide spacing to prevent mutual shading and tiller shoot mortality (Srivastava and Rai, 2012).

### Accumulation of reactive oxygen species

**Figure 5:**
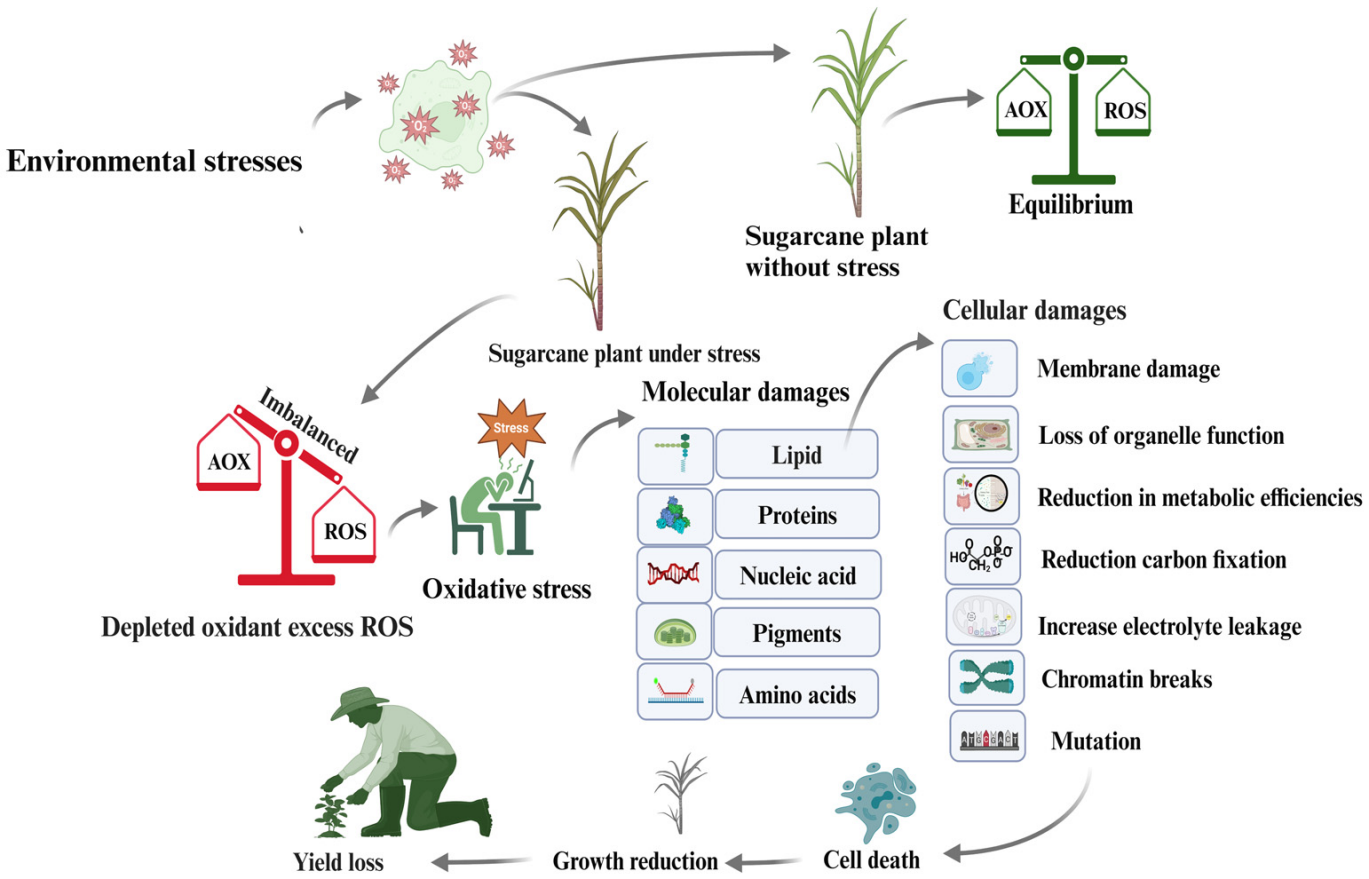
Mechanism of ROS accumulation in sugarcane plant cells as a consequence of oxidative stress, leading to reductions in sugar content and sugarcane yield.

Environmental stressors, such as light, heat, salt, drought, nutrient shortage, and pathogen invasion, cause plants to produce ROS, primarily in the mitochondria, chloroplasts, and peroxisomes. These ROS produce hydrogen peroxide, superoxide radicals, and hydrogen radicals. Under stressful conditions, ROS create an imbalance between antioxidants and other biomolecules, causing oxidative stress. Lipid peroxidation occurs when O_2_ molecules enter plastid membranes, leading to membrane fragmentation and altering cell membrane properties (Huang et al., 2012). This damage can cause cell death, reduced crop yield, and enzyme or protein denaturation. Sucrose-metabolizing enzymes are crucial for sucrose accumulation and yield formation, but ROS can inhibit or denature these enzymes, thereby affecting sugar production. Increased ROS levels during stress trigger the synthesis of post-translation protein modifiers, such as SUMO proteins, and the antioxidant enzyme superoxide dismutase (SOD), which can reduce environmental stress (Ogbaga et al., 2020). Plants show normal activity when ROS and antioxidants are balanced; however, an imbalance can cause oxidative stress, leading to molecular damage, membrane instability, hydrogen peroxide increase, improper function, cellular death, and ultimately, yield decline (**Figure 5**).

### Plant adaptation to environmental stress

Plants are classified based on temperature tolerance into three groups: heat-sensitive, relative heat-resistant, and heat-tolerant species. They develop survival strategies to cope with environmental stresses. The two mechanisms identified for water loss in plants are short-term avoidance through morphological and phenological adaptations and long-term mechanisms, such as stomata closure, lipid composition changes, and leaf orientation (Srivastava et al., 2012). During the period of rapid development, plants are vulnerable to several environmental stressors. Crops are protected by agricultural practices and tolerance mechanisms. Plants accumulate various substances, including metabolites, heat shock proteins, osmolytes, and antioxidants, under different environmental stresses.

### Accumulation of metabolites and osmolytes

Plant metabolites are essential for growth, survival, and development and act as defense mechanisms under environmental stresses. They detect threat signals and increase metabolite production, which is influenced by developmental stages and physiological conditions and plays a pivotal role in interactions, such as mutualism and antagonistic relationships (Chen et al., 2019). Plant hormones, such as abscisic acid (ABA), jasmonic acid (JA), polyamines, and salicylic acid (SA), are essential for defense against stresses. Hormones are produced in plant roots to regulate plant development and respond to drought stress; notably, abscisic acid production increases for stomatal closure and opening (Sage et al., 2013). ABA controls two-thirds of genes under drought stress, increasing calcium ions and triggering membrane Ca^2+^ permeable channels. It plays three major roles in plant growth: protecting leaf growth rate fluctuations, controlling stomatal movement, and enhancing tissue hydraulic conductivity (Xu et al., 2018). The up-and-down regulation gene (*ERA1*) has been successfully manipulated to address drought stress (Daszkowska-Golec et al., 2018). Another important stress indicator is proline accumulation. Amino acids are essential for plant and cell survival under environmental stress, and proline accumulation is 100 times higher under environmental stress conditions than under normal conditions (Santos et al., 2019). In transgenic sugarcane plants, maximum proline accumulation occurs under water stress conditions (Li et al., 2018). P5CS, an enzyme in plants, catalyzes proline biosynthesis, provides carbon and nitrogen, maintains cytosol pH, prevents protein denaturation, and sustains cell redox levels under stress conditions (Maggio et al., 2002). This accumulation is observed in response to cells damaged by ROS, suggesting that maximum proline accumulation is an adaptive response to cell damage or an osmoprotectant. These findings indicate the importance of developing transgenic sugarcane cultivars resistant to environmental stress by modifying the ABA regulatory network and proline pathway and provide a new avenue for sugarcane molecular breeding. Sugarcane is subject to several environmental stressors, mostly due to its prolonged growth season. Thus, developing multiple stress-resilient cultivars is crucial, given that drought stress can enhance sugarcane susceptibility to pests and diseases.

## Stress-related genes and proteins

Heat shock proteins (HSPs) are essential for plant survival under heat-stress conditions. The five classes of HSPs in plants are HSPs 60, HSPs 70, HSPs 90, HSPs 100, and small heat shock proteins. The upregulation of HSP70 enhances water content, membrane permeability, and photosynthetic rate (Murphy, 2011). HSPs also function as molecular chaperones to protect enzymes and proteins from denaturation and direct thermal stress transcriptional factors (Augustine et al., 2015). Plants have developed stress response mechanisms to preserve proteins in complementary and sometimes overlapping ways (Wang et al., 2016). For instance, under abiotic stress, HSPs, such as the *Arabidopsis* ligase gene (*AtSIZ1*), are essential for protein refolding. The overexpression of the rice gene *OsSIZ1* in cotton can improve fiber output and resilience (Mishra et al., 2017). SUMO1 and SUMO2 are posttranslational modifier proteins influenced by environmental stresses, such as high temperature, chilling, oxidative stress, and drought (Rabara et al., 2014). Garcia Tavares et al. (2018) altered the expression of ScGAI in sugarcane and postulated that the overexpression of the excessively tolerant to salt1 (*OTS*) gene may be important for SUMOylation and the reduction of *ScGAI* during drought stress. The overexpression of the *ScGAI* gene led to decreased carbon supply to the stem, increased tiller numbers, and hindered sugarcane growth. The alteration in the *ScGAI* gene is crucial for growth and development, tillering, plant height, and carbon allocation. Late embryogenesis abundant (LEA) proteins, encoded by the ubiquitin group of stress-targeting genes, are present in plant seeds and sugarcane leaf tissues. Maximum water bonding occurs during drought conditions because of the hydrophilic nature of this protein (LEA). Dehydrin proteins, specifically group II LEA proteins, enhance water holding capacity and ions on cell surfaces, leading to higher relative water content in drought-resistant plants (Chen et al., 2019). The small basic intrinsic proteins (SIPs), NOD26-like intrinsic proteins (NIPs), tonoplast intrinsic proteins (TIPs), and plasma membrane intrinsic proteins (PIPs) comprise the stress-responsive protein subfamily known as AQP. The AQP family proteins (PIP1, NIP, and SIP isoforms) showed upregulation in root tissues; however, PIP2 isoforms were expressed in leaves under stress conditions (Afzal et al., 2016). After 21 days of water stress, *ShPIP2;1, ShPIP2;5*, and *ShPIP2;6* were upregulated; after soil rehydration, they were downregulated, suggesting that *PIP2* controls crop water status (Ferreira et al., 2017). Overexpression of *PP2* proteins in *Arabidopsis* leads to sucrose accumulation under stressful conditions, while overexpression in *Eucalyptus globulus* is linked to cold temperatures (Feltrim et al., 2021). Additional investigations are required to comprehend the role of AQPs in sugarcane under environmental stresses and develop varieties with maximum sucrose accumulation and stress tolerance. The study of the SUMOylation mechanisms in sugarcane can lead to the development of new crop improvement markers that regulate physiological functions and drought resistance.

## Strategies to mitigate environmental stress

Strategies should be implemented in accordance with specific characteristics to adapt to environmental stressors. Cultivating resistance varieties can help alleviate oxidative stresses caused by biotic and abiotic factors. Sugarcane cultivars exhibit specific variability in stress tolerance, with some varieties showing superior performance under various environmental stresses (Bunyatta et al., 2021). Hence, scientists should continue breeding sugarcane cultivars that are more resistant to environmental stresses. Irrigation scheduling using canopy temperature sensors can effectively mitigate the negative effects of temperature and drought stress on plant growth. Exogenous application of synthetic and natural plant growth regulators is a crucial and efficient agronomic strategy to mitigate the negative effects of biotic and abiotic stresses. Scholars have proposed adaptation and mitigation strategies to address the impact of climate change, and these include the development of thermotolerant, diseased resistant, and drought-tolerant cultivars, improving drainage and irrigation efficiency, and enhancing cultural and management practices. Sugarcane breeding projects primarily employ hybridization and selection to produce new recombinant clones with high yield, sugar content, and stress resistance (Kumar et al., 2023). Researchers use molecular biology, biotechnology, tissue culture, and transformation to improve sugarcane selection and breeding efficiency to obtain varieties that are resilient to environmental challenges. Breeders have traditionally focused on developing high-yielding varieties but have now prioritized stress resistance due to shifting climatic conditions. They are developing new varieties against biotic and abiotic stresses by using molecular and conventional breeding methods. Conventional breeding has produced numerous sugarcane-resistant and high-yielding varieties globally for the past five decades. However, the complex sugarcane genome, with multiple genes for each character, introduces unpredictable traits and is a time-consuming and costly process.

## Modern or molecular breeding

### Sugarcane resistance to abiotic stresses

Transgenic methods, such as particle bombardment, cell electroporation, and *Agrobacterium tumefaciens*, are used in sugarcane molecular breeding to transfer desired traits and improve crop yield. Gene transfer has enhanced traits in sugarcane, including water stress, herbicide resistance, pest resistance, disease resistance, and maximum sucrose accumulation (Suprasanna et al., 2011). Although regulatory barriers are available to field evaluation of transgenic plants, transgenic plants have a high chance of overcoming environmental stresses. Most of the sugarcane transgenic lines have been screened for thermotolerance, drought, and salt resistance under *in vitro* conditions due to regulatory barriers (Narayan et al., 2021). Furthermore, limited data are available on the growth of transgenic sugarcane plants in natural environments (De Souza et al., 2019). Three transgenic sugarcane cultivars that are resistant to drought stress were recently approved by the Ministry of Agriculture and introduced in Indonesia (James, 2013). These GM varieties are cultivated on around 1315 hectares of land. Brazil also approved the first commercially available genetically modified sugarcane cultivars resistant to the insect known as sugarcane borer (Phillips, 2017; International Service for the Acquisition of Agri-biotech Applications (ISAAA), 2018). Recently, China used molecular breeding to develop a transgenic sugarcane (FN95-1702) crop with drought tolerance and high yield characteristics (Xiao et al., 2022). **Table 1** enumerates some significant gene activities conducted to enhance tolerance to environmental stressors and obtain cultivars with high yield or high sucrose accumulation.

**Table 1:** Transformed potential gene functions against abiotic stresses.

Gene name	Environmental stresses	Functions	References
*SsGTEL3a*	Drought	Response to abiotic stress	(Jiang et al., 2023)
*BRK1*	Drought	Response to drought stress and improved physiological parameters	(Narayan et al., 2023)
*ScDIR*	Drought	Response to drought stress	(Li et al., 2022)
*EaEXPA1*	Drought	Maintain the thermostability of the cell and chlorophyll content	(Narayan et al., 2021)
*Transcription factors*	Cold	Response to cold stress	(Rehman et al., 2021)
*ShGPCR1*	Cold, drought and salt	Calcium (Ca^2+^) enhance in sugarcane cells	(Ramasamy et al., 2021)
*ScAOC1*	Disease resistance, cold	Enhanced in the presence of SA, ABA, PEG, cold stressors and smut resistance	(Sun et al., 2020)
*AtBBX29*	Drought	Increase antioxidants and protect photosynthetic machinery	(Mbambalala et al., 2021)
*Dehydrins (21,23* and 27 kDa*)*	Heat stress	Enhance the plant’s capacity to retain ions and water on its cell surface	(Wahid and Close, 2007)

### Sugarcane varieties resistant to diseases

Disease and pests are the main biotic stressors that affect sugarcane yield and sucrose formation. Disease control in sugarcane production systems involves disease-resistant varieties, disease-free planting material, proper farm management, and strict quarantine protocols. More than 100 pathogens contribute to sugarcane diseases and affect production worldwide. A diverse range of disease resistant varieties is crucial for successful breeding (Govindaraju et al., 2019). Selection and screening of sugarcane for disease resistance is one of the most crucial phases in a breeding program. Sugarcane breeders face challenges in introducing resistance genes against all pathogens simultaneously through conventional breeding, making commercial cultivars vulnerable to multiple pathogens (Cursi et al., 2022). Many high-yielding clone breeding programs were not commercially released due to increased susceptibility to certain diseases (Babu et al., 2021). Molecular breeding techniques are being investigated to provide commercial clones with long-lasting disease resistance and improved agronomic performance. Sorghum Mosaic Virus (SrMV) and Sugarcane Mosaic Virus (SCMV) are two major viral diseases that significantly affect sugarcane yield. Both diseases have been documented globally. Several genetic transformation approaches have been used to generate resistant plants because of the destructive consequences and extensive dissemination of these viruses. For instance, scientists successfully developed virus-resistant varieties (Q95, Q153, and Q155) against the sugarcane mosaic virus (SCMV) *CP* gene through microprojectile bombardment (Joyce et al., 2014). Another virus-resistant transgenic variety (CP65-357 and CP72-1210) was developed against the Sorghum mosaic virus (SrMV) (Ingelbrecht et al., 1999). The transgenic variety Q124 was developed against the Fiji disease virus (FDV). Transgenic lines demonstrated enhanced FDV resistance in glasshouse trials, but their molecular phenotypes are not fully aligned with PTGS-based resistance mechanisms (McQualter et al., 2004). The list of transgenic sugarcane disease-resistant varieties is provided in **Table 2**.

**Table 2:** Transformed potential genes against target diseases in different sugarcane varieties.

Transgene name	Target disease	Variety	References
***ScCAT1***	Whip smut	ROC22 and YT93-159	(Wu et al., 2023)
***CP***	SCMV	SPF-234	(Aslam et al., 2018)
***Chitinase class-ii***	Red rot	S2006SP-93	(Tariq et al., 2018)
***ß-1,3 glucanase***	Red rot	CoJ83	(Nayyar et al., 2017)
***CP***	SrMV	ROC22	(Guo et al., 2015)
***Segment 9 of ORF 1***	FDV	Q124	(McQualter et al., 2004)
***Bru1***	Rust	R570	(Asnaghi et al., 2004)
***Glucanse and Chitinase***	Brown rust	B4362	(Enriquez et al., 2000)

### Sugarcane varieties resistant to insect pests

Insect pests affect sugarcane yield, reduce sugar content, and potentially lead to economic loss worldwide (Iqbal et al., 2021). Genetic transformation technology has revolutionized plant genetic engineering for pest protection by transferring genes from plants, pests, and bacteria (Gosal and Wani, 2018). For instance, protease inhibitors and lectins (plant-derived insecticidal proteins) are efficient at inhibiting insect development and reproduction. Deng et al. (2008) utilized genes, such as *avac, skti, sbbi*, and *gna*, in genetic transformation programs to develop insect resistance in sugarcane. Transgenic sugarcane plants with the snowdrop lectin gene showed improved antibiosis against sugarcane grub larvae but only gained 20.6% of weight gain compared with non-transgenic control plants (Allsopp et al., 2000). Similar results were reported on greyback sugarcane beetles’ larvae (Nutt et al., 2001). When soyabean genes *skti* and *sbbi* were transformed into sugarcane, sugarcane borers were significantly reduced (Falco and Silva-Filho, 2003). Another study on aprotinin-transgenic sugarcane revealed that top borer larvae fed on these plants had weight reductions of up to 99.8% (Christy et al., 2009). Researchers developed transgenic sugarcane lines overexpressing *CaneCPI-1* and tested their resistance against sugarcane billbug larvae, leading to slight damage to the transgenic plants (Punithavalli, 2022). *Bacillus thuringiensis* (*Bt*) produces highly toxic insecticidal proteins, which are used in transgenic plants to control insect pests due to their efficiency (Baranek et al., 2017). The success of *Bt* crops in agriculture leads to increased interest in controlling sugarcane pests, particularly stem borers. Transgenic sugarcane with the *cry1Ab* gene was developed, and various *Bt* genes have been shown to improve resistance to various *Lepidopteran* pests (Arencibia et al., 1997). Transgenic sugarcane expressing *Bt* toxins, particularly *vip3A* toxin, has demonstrated effectiveness against various pests, with a 100% mortality rate against sugarcane shoot borer in field tests (Riaz et al., 2020). The *vip3A* gene might be used in gene pyramiding with other *Bt* toxins to enhance and extend resistance (Cristofoletti et al., 2018). Wang et al. (2023) found that the sugarcane gene *ScWIP5* toxicity led to high mortality and weight loss in armyworms, high jasmonic acid levels, and low levels of digestive enzymes in the insect gut. In 2017, the transgenic sugarcane variety CTB141175/01A, which expresses the *cry1ab* gene, was first commercially released as a result of the successful suppression of stem borers in sugarcane by the application of *Bt* toxins. Two sugarcane transgene lines (CTC91087-6 and CTC93209-4) with the *cry1Ac* gene have been released in Brazil (ISAAA, 2021). Several transgenic lines have been developed after inserting resistant genes into sugarcane plants (**Table 3**).

**Table 3:** Transformed potential genes against target pests in different sugarcane varieties.

Gene Name	Target pest	Variety	References
***Vip3A***	*Chilo influscatellus*	CPF-246	(Riaz et al., 2020)
***Cry1Ac***	*Sesamia Cretica*	GT54-9 (C9)	(Dessoky et al., 2021)
***Cry1Ab-Cry1Ac***	*Scrippphaga excerptalis*	Bululawang	(Koerniati et al., 2020)
***Cry1ac***	*D.sachharalis*	Event CTC91087-6	(Gianotto et al., 2019)
***Cry1Ab***	*D.sachharalis*	Event CTC175-A	(Cristofoletti et al., 2018)
***Cry1Ab-Cry2Ab***	*D.sachharalis*	SP-803280	
***Cry2A***	*C.sachhariphagus,S.nivella*, *C. schistaceana and S.inferens*	ROC22	(Gao et al., 2018)
***Cry1Ac***	*D.sachharalis*	FN15 and ROC22	(Zhou et al., 2018)
***Cry1Ab***	*D.sachharalis*	ROC22	(Wang et al., 2017)
***CaneCPI-1***	*Sephenuphorus levis*	SP80-185	(Schneider et al., 2017)
***Cry1Ab***	*D.sachharalis*	LK 92-11	(Islam et al., 2016)
***Cry1Ac***	*D.sachharalis*	FN15	(Gao et al., 2016)

## A novel approach to sugarcane enhancement: Endophyte biocontrol applications

Endophytic microbes, residing within plant tissues, play a crucial role in plant growth, development, and survival. They can be found in various parts of plants and serve as biofertilizers, biological control agents, and inducers of tolerance (Burragoni and Jeon, 2021).

### Endophytes as plant biocontrol agent

Endophytes improve plant growth and enhance agricultural productivity through hormone development, water absorption, and increased access to nutrients, such as nitrogen, phosphorous, potassium, zinc, and iron (Baron and Rigobelo, 2022). Endophytic fungus (*Fusarium oxysporum*) can change hormonal pathways in wheat plants, increasing biomass both above and below the ground as well as the growth of the roots and stem length, and increasing the germination rate. Four taxa of endophytic fungi including *Aspergillus*, *Penicillium*, *Fusarium*, and *Trichoderma*, were recently identified in sugarcane plants. In sugarcane bud-sett, the application of *Trichoderma* sp. and *Penicillium* sp. showed favorable effects on plant height, stem diameter, and leaf size (Sektiono et al., 2023). Many studies found that endophyte application on sugarcane improved productivity against environmental stressors. The combination of *Azospirillum brasilense* + *Pseudomonas* fluoresces promotes plant growth and increases sugarcane stem productivity by 42% (Fernandes et al., 2023). Based on this study, coupling two different bacterial species can boost productivity, decrease phosphate fertilization, and increase sugarcane yield. Another study on nitrogen-fixing bacteria (two *Pseudomonas* strains; *P. koreensis* CY4 and diazotrophic CN11) reported increased levels of antioxidant enzymes (POD, SOD, and CAT), glucanase, and phytohormones (abscisic acid and cytokinin) (Singh et al., 2023). Furthermore, *Bacillus subtilis* B9 application on sugarcane had similar results (Di et al., 2023). Hence, bacterial endophytes or plant growth-promoting bacteria are potential biofertilizers for sugarcane crops.

### Endophytes promote plant resistance to abiotic stresses

Throughout their life cycle, crops are subject to a variety of environmental challenges, such as salinity, drought, high temperatures, heavy metal toxicity, and insufficient nutrients. These stressors, which result in ion toxicity, photosynthetic suppression, membrane integrity loss, or an increase in ROS formation, can substantially decrease crop yield and plant growth. Additionally, it can mitigate environmental impacts by promoting stress-tolerant plant responses or directly producing biochemical substances that mitigate stress (Lata et al., 2018). For instance, endophytic fungi or bacteria can synthesize or stimulate the synthesis of organic solutes (proline), hormones, secondary metabolites, and antioxidant enzymes (SOD, CAT) in plants (Tyagi et al., 2022). In the absence of abiotic stress, inoculating *tritordeum* and perennial ryegrass with endophyte (*Diaporthe*) improved plant growth, nutritional content (N, Ca, Mg, Fe), and auxin production. Furthermore, endophytic fungi or bacteria mitigate salinity stress in plants by promoting plant synthesis, proline, gibberellin, and auxin accumulation, and nutrient uptake by roots. A recent study on 43 bacterial endophytes from the sugarcane variety (Yunzhe 99–91) demonstrated that *Bacillus subtilis* (*B9*) promoted phytohormone level, macronutrition (N, P, and K), photosynthesis rate, and root development of sugarcane (Di et al., 2023).

### Endophytes as biological agents of pest and disease management in crops

Biotic stressors (pathogens, animals, and weeds) result in 20%–40% crop losses yearly, of which 10%–15% are pathogen-specific losses (Mohammad-Razdari et al., 2022). Bacterial and fungal endophytes can help prevent crop losses by indirectly inducing defenses against pathogens, directly attacking pathogens through mycoparasitism and nematode parasitism, and competing for plant space and nutrients (Poveda et al, 2022). Endophytes can infect insect tissues directly or create insecticidal chemicals; they can also indirectly trigger defenses in the host plant and induce insect repellent or antifeedant responses (Poveda et al, 2021). As an example, *Trichoderma* species (*T. asperelloides and T. lentiforme*) can effectively control the cotton pathogen *Sclerorinia sclerotiorum* by inhibiting its growth and *mycoparasitizing* the sclerotia formed by the pathogen (De Silva et al., 2019). Endophytes (*Hypocrea* and *Trichoderma*) inoculated in the roots of tomato and bean plants reduced oviposition and adult longevity in greenhouse whiteflies, which led to improved host plant defenses (Parada et al., 2022). Agbessenou et al. (2022) discovered a mechanism using *Trichoderma asperellum* to mitigate tomato leafminer damage. *T. asperellum* initiates a defensive response by releasing methyl salicylate (a volatile substance) that repels *T. absoluta’s* herbivory attack. Favaro et al. (2012) found that the application of *Epicoccum nigrum P16* increased the root biomass of sugarcane endophytes and controlled their pathogenicity. Further, the endophyte *T. viron* from sugarcane can inhibit the pathogen or induce systemic resistance (Romao-Dumaresq et al., 2016). Moreover, several endophytes may be antagonistic to sugarcane smut, including *Ramichloridium, Alternaria, Sarocladium, Epicoccum*, and *Exophiala* species (Chen et al., 2019). **Table 4** shows all fungal, bacterial, and viral diseases, their casual organisms, and their control by endophytes. Additionally, endophytes play an important role in many important agricultural crops under harsh environmental conditions. Endophytes produce metabolites, antioxidants, and, most importantly, exhibit antagonistic activity against pathogens. **Table 5** presents examples of effective endophyte applications for different crop species.

**Table 4:** Casual organisms and biocontrol agents of sugarcane diseases.

Sugarcane Fungal Diseases			
Diseases Name	Casual Organism	Endophytic control agents	References
**Red rot**	*Colletotrichum falcatum*	*B. velezensis YC89 strain*	(Xie et al., 2023)
**Sugarcane Smut**	*Sporisorium scitamineum*	*Trichoderma, Pseudomonas, or Bacillus spp, Pseudomonas aeruginosa*	(Singh et al., 2021a)
**Pokkah boeng**	*Fusarium sacchari*	*T. harzianum, T. hamatum, T. viride, A. flavus, and A. niger*	(Srivastava et al., 2019)
**Pineapple disease**	*Ceratocystis paradoxa*	*Trichoderma harzianum*	(Talukder et al., 2007)
Sugarcane Bacterial Diseases			
***Red stripe***	*Acidovorax avenae subsp. avenae*	*Amycolatopsis strain*	(Guerrero et al., 2022)
***Ratoon stunting disease***	*Leifsonia xyli*	*Diazotrophic*	(Carneiro et al., 2021)
***Leaf Scald disease***	*Xanthomonas albilineans*	*Gluconacetobacter diazotrophicus*	(Arencibia et al., 2006)
Sugarcane Viral diseases			
**Sugarcane mosaic virus (SCMV)**	*Mosaic virus*	*T. harzianum and M. anisopliae*	(Kiarie et al., 2020)

**Table 5:** Effects of the consortium application of endophytes on different crop species.

Plants	Consortium endophytes Application	Effects	References
**Tomato**	*Bacillus velezensis,Bacillus megaterium and Herpaspirillum huttiense*	Declined MDA and H_2_O_2_ content while increased the antioxidant enzymes activities	(Abbas et al., 2024)
**Rice**	*Azotobacter, Bacillus, Enterobacter, and Xanthobacter,Anabaena variabilis, Tolypothrix tenuis, Nostoc muscorum, Aulosira fertilissima*	Enhanced the number of weight grain yield, number of productive tillers and resistance to pathogens by producing phenolic compounds	(Doni et al., 2022; Prihatiningsih et al., 2023)
**Chilli**	*Erwinia persicina EU-A3SK3, Halomonas aquamarina EU-B2RNL2, and Pseudomonas extremorientalis EU-B1RTR1*,	Increase the phenolic compounds, chlorophyll content, biomass and fruit per plant	(Devi et al., 2022)
**Robusta coffee**	9 *bacterial isolates of genus Bacillus and Pseudomonas*	Suppressed the nematodes (*P. coffeae*) improved the crop growth	(Asyiah et al., 2020)
**Wheat**	*Bacillus* sp. *MN54 + Trichoderma* sp. *MN6*	Increase chlorophyll content, leaf areas and crop productivity	(Muhae-Ud-Din et al., 2018)
**Brassica**	*Pseudomonas fluorescence strains,rhizophoric P. fluorescence F113*	Increased seeds and oil yield	(Lally et al., 2017)

## Relationships between host plants and endophytes

**Figure 6:**
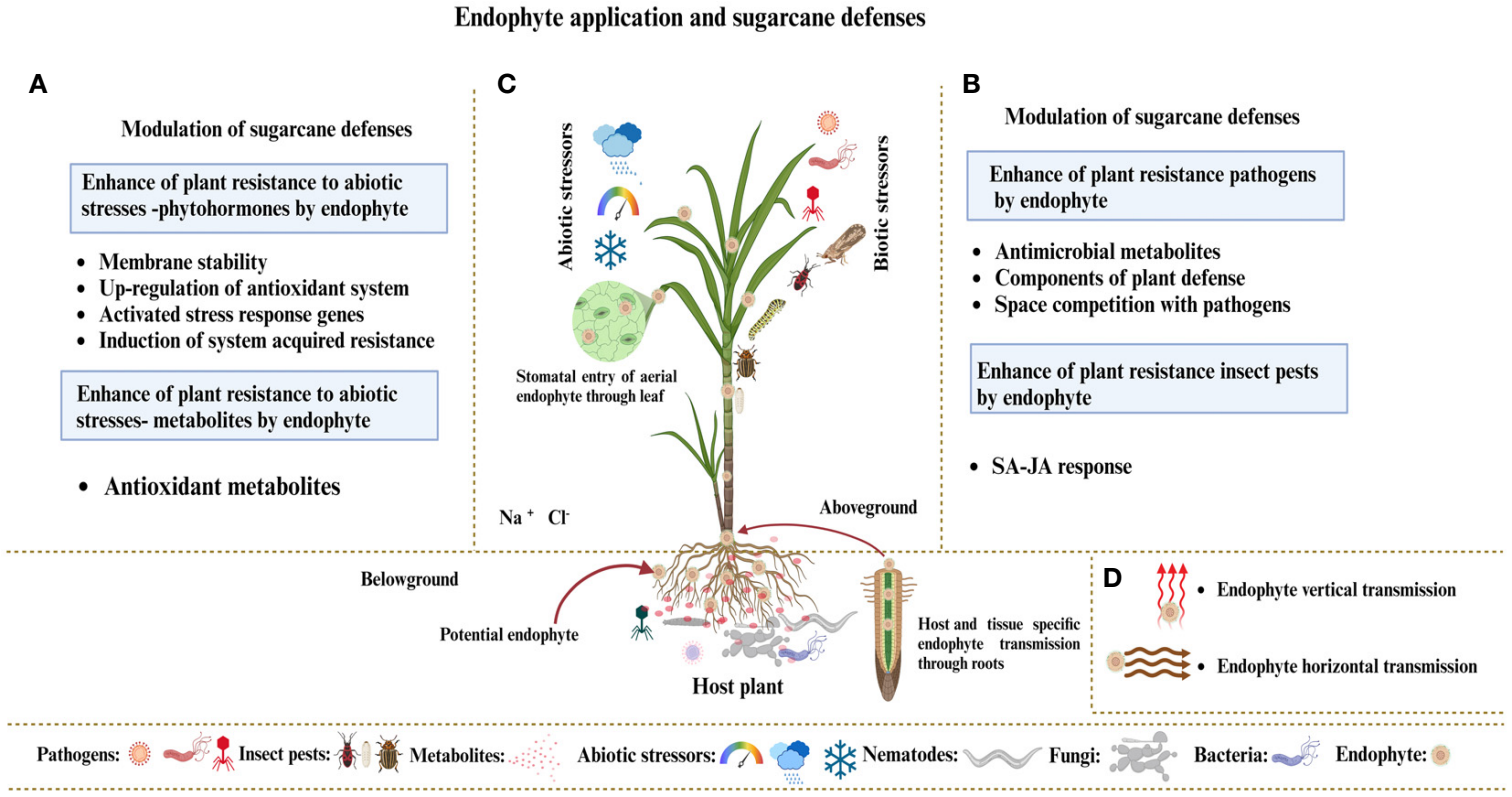
**(A)** Hypothetical model demonstrating how endophyte colonization affects sugarcane plant defenses against various abiotic stresses, such as heat, cold, humidity, salt, and drought. **(B)** Endophytes boost plant immunity by producing protective metabolites and modifying the phytohormone mechanism under biotic stressors, such as pathogens and insect pests. **(C)** Endophytes exist in various organs of plants, including leaves, stems, and roots. Endophytes enter the plant via roots, xylem tissues, and leaf stomata. **(D)** Endophytes typically use two transmission methods: vertical transmission from parent plants to their offspring and horizontal transmission between plants through spores, hyphal fragmentation, or biotic or abiotic dispersion agents, allowing for the spread of different host plants.

Endophytes live inside plant organs and can colonize tissues without endangering their host. Research mainly focuses on endophytic bacteria or fungi, which benefit plants and their interacting partners. Factors affecting the development of endophytic fungus populations include soil type, plant species, habitat, and microorganisms (Le Van et al., 2017). Geographical and climate variables significantly affect the root endophytic fungal populations of *Microthlaspi* because endophytes in plant roots exhibit stochastic assembly processes due to growth habits and dispersion capacities (Powell et al., 2016; Glynou et al., 2016). Root exudates are essential in determining the composition and assembly of the rhizosphere microbiome and represent all plant metabolites that reach the rhizosphere (Sasse et al., 2018). Root exudates consist of various molecules, including sugars, polysaccharides, and proteins, which have different molecular weights (Busby et al., 2017). These substances exert significant chemical signals within plants and the rhizosphere microbiome. Soil microorganisms initiate contact and communication by recognizing chemical signals released by plants (Ray et al., 2019). Plants form symbiotic relationships with soil microorganisms, and some of which can live within their tissues, leading to endophytic interactions with their hosts. The endophytic fungus first sticks to the root surface and forms structures resembling appressoria (Yedidia et al., 1999). After sticking, the fungi break through the outer layers of the root systems to reach the interior plant tissues. Endophytic fungi regulate host genes, phenotypes, and metabolism to alter nitrogen metabolism, improve photosynthesis, and resist insect pests, enabling them to live asymptomatically and benefit their host plants. Plants can detect microorganism signals and respond appropriately by triggering their defense mechanisms, despite the positive effects of the plant–endophyte relationship (Zipfel and Oldroyd, 2017). Plants rely on innate immunity to recognize the molecules of microbial signals, which results in the development of two different defense systems: (i) plants utilize cell surface-localized pattern recognition receptors (PRRs) to identify microbe-associated molecular patterns (MAMPs), thereby triggering MAMP-triggered immunity (MTI); and (ii) plants utilize internal sensors to detect bacteria-generated chemicals, known as “effectors,” and then activate effector-triggered immunity (ETI) in response (Mendoza-Mendoza et al., 2018). Endophytes enter the plant either from the root via xylem tissue or from the leaf tissue via stomata. Endophytes adopt two modes of transmission: (i) vertical endophytes transfer from parent plants to offspring (Gagic et al., 2018) and (ii) endophytes in surface tissues are horizontally transmitted among plants through spores, hyphal fragmentation, or biotic or abiotic dispersion agents, allowing for the spread of various host plants. Endophytes exist in different plant parts, including roots, stems, and leaves. The current research focus is on the application of endophytic fungi as biocontrol agents for sustainable agriculture (**Figure 6**).

## Endophyte application strategy

### Endophytic consortium: an alternative to transgenic co-expression of multiple genes

Different endophytic strains play different roles in plants. A single endophyte may not have all the desired traits, such as induction of plant defense response, antimicrobial and metabolite productions mandatory for increasing sugarcane crop yield, enhanced synthesis of secondary metabolites, and defense against environmental stressors in plants. Furthermore, the limited efficacy of single-strain inoculation in various geographical areas and field situations constrains its application (Compant et al., 2019). Distinct agronomic traits or mechanisms must either complement one another or be combined to achieve various or multiple benefits, such as improved plant development; related strategies include enrichment of secondary metabolites, resistance to environmental stress, and pathogen biocontrol. Additionally, strains with the same method of action may be included in endophyte consortia but should sustain various kinds of environmental challenges or are adaptable to various crop varieties. Many studies showed that the application of the endophyte’s consortium enhanced plant growth and development. Hence, in natural environments, consortium application is a more promising strategy than single-strain application (De Vrieze et al., 2018). Endophytes utilize distinct metabolic pathways or mechanisms to influence host metabolism at the molecular level, specifically through gene expression. Combining multiple endophytes with distinct methods can enhance gene upregulation, leading to improved secondary metabolite production (Ray et al., 2019). These endophyte consortia are a more suitable option than transgenic plants, which overexpress or co-express several genes. Several experiments have developed methods for introducing different endophytes into various plant tissues or targets (Pandey et al., 2018; Ganie et al., 2022). For instance, the application of an endophytic consortium improved crop production (Tripathi et al., 2020) and secondary metabolite production in different varieties of *Catharanthus roseus* (Singh et al., 2021b). The application of seven endophytic consortia enhanced maytansine biosynthesis, which is a potential antifungal agent (Pitakbut et al., 2022). Ganie et al. (2022) recently examined the use of endophytes, especially their consortia, to obtain rice plants resilient to biotic and abiotic stress. Scholars have discussed how various endophytes target different cellular components to provide stress tolerance to rice plants. A consortium of endophytes with diverse stress tolerance and yield enhancement strategies is the most effective method for enhancing sugarcane plant performance under field conditions (**Figure 6**).

## Concluding remarks and future perspectives

Abiotic and biotic stresses considerably affect sugarcane yield and sucrose production, and future research on soil nutrition, stress resistance, pests, and diseases is much needed. Genetic engineering can create environment-friendly sugarcane varieties by reducing harmful effects. Biotechnology has been used to develop sugarcane events with resilience, but few global efforts have successfully introduced transgenic varieties for commercial release. Despite genetic advancements, transgenic sugarcane lines are not commercialized because of regulatory concerns and uncertainty about their field performance. Brazil and Indonesia recently approved a transgenic sugarcane variety for commercial cultivation. Other sugarcane-producing countries have contributed to sugarcane improvement programs worldwide to combat environmental challenges and food insecurity. The important role of endophytes in sustainable sugarcane productivity under environmental stresses cannot be overlooked. The current study explores a novel approach involving the consortium application of endophytes to sugarcane plants and focuses on its primary advantage of increased plant adaptability to biotic and abiotic stressors. Most importantly, endophytes are an alternative to overexpressing multiple genes in plants. The endophyte consortium triggers the genes of primary and secondary metabolites to improve crop yield and survival under different environmental stresses. In this regard, numerous reports have indicated that endophytic fungi support host plants in combating biotic stressors from pathogens. The main threats to sugarcane plants are infectious diseases, which also lead to large financial losses for the sugarcane industry. The development of endophytic fungi as biocontrol agents for sustainable agriculture is now centered on their use. Endophytic fungi (*Aspergillus, Penicillium*, and *Fusarium*) produce antimicrobial compounds that help their host plants resist infections. Endophytes exist in various organs of plants, including leaves, stems, and roots. Antifungal metabolites found in root endophytes defend roots from pathogen infection and may act as a first line of defense for early seedlings. Additionally, endophytes can induce plants to overproduce immune or antibacterial chemicals. For instance, the endophytic fungus *Trichoderma hamatum* can cause plants to overproduce defensive enzymes and PR proteins as well as endogenous salicylic acid, which can strengthen the plants’ ability to fight against the pathogen. In plant leaves, host defense genes are upregulated by the endophytic fungus *Colletotrichum tropicale*, possibly contributing to resistance against pathogen damage. A pathogen (*Pythium myriotylum*) causes red rot disease in tomato plants at the vegetative stage. *Beauveria bassiana*, an endophyte, can control the red rot disease; it also helps plants adapt to environmental stressors, facilitates soil nourishment from the soil to the roots, and controls plant growth and development. The discovery of ancient fungal hyphae (fossilized fungal hyphae) raises the possibility that terrestrial plants require endophytic association to survive abiotic stresses, such as drought, salinity, and high temperatures. Endophytic fungi facilitate hormone production and nutrient uptake, helping host plants adapt to and grow in an abiotic environment. Endophytes reduce the reactions of plants to abiotic stresses by controlling the hormonal balance and promoting systemic stress tolerance. For instance, bacterial endophyte (*Bacillus subtilis*) induces plant growth by promoting plant growth hormones abscisic acid (ABA) and indole acetic acid (IAA), resulting in enhanced root and shoot mass in pea plants under salt stress. Plant hormones, such as salicylic acid, inhibit lipid peroxidation and enhance cell membrane thermostability in plants. Many endophytes are isolated from sugarcane, such as *Aspergillus, Penicillium, Fusarium*, and *Trichoderma*, which affect sugarcane shoot length, increase stem diameters, and promote leaf growth. A previous study reported that the endophyte *Pseudomonas aeruginosa* (*B18 strain*) found in sugarcane showed resistance to the sugarcane smut disease pathogen (*Sporisorium scitamineum*) in a susceptible cultivar (Yacheng 71–374) and was effective against *Trichoderma* isolated from sugarcane. Endophytes could be considered antagonistic to sugarcane smut and crucial in reducing biotic and abiotic stresses. The application of endophytes can enhance sugarcane yield and sugar content by combating environmental stresses. This review provides new insights for the application of consortium endophytes to increase crop yield and improve plant health.

## Author contributions

FM: Conceptualization, Formal analysis, Methodology, Writing – original draft, Writing – review & editing. ZC: Conceptualization, Formal analysis, Supervision, Writing – review & editing. SZ: Methodology, Validation, Writing – review & editing. YG: Formal analysis, Methodology, Writing – review & editing. WC: Data curation, Writing – review & editing. LP: Investigation, Validation, Writing – review & editing. YW: Software, Visualization, Writing – review & editing. WW: Investigation, Validation, Writing – review & editing. BY: Funding acquisition, Resources, Supervision, Writing – review & editing.
