# Corrigendum: Factors affecting the production of sugarcane yield and sucrose accumulation: suggested potential biological solutions

**DOI:** 10.3389/fpls.2024.1499021

**Published:** 2024-10-18

**Authors:** Faisal Mehdi, Zhengying Cao, Shuzhen Zhang, Yimei Gan, Wenwei Cai, Lishun Peng, Yuanli Wu, Wenzhi Wang, Benpeng Yang

**Affiliations:** ^1^ National Key Laboratory for Tropical Crop Breeding, Institute of Tropical Bioscience and Biotechnology, Chinese Academy of Tropical Agricultural Sciences, Haikou, China; ^2^ Sanya Research Institute, Chinese Academy of Tropical Agricultural Sciences, Sanya, China

**Keywords:** environmental stresses, biocontrol agents, resistance genes, sugarcane, sucrose accumulation, tolerant varieties, yield

In the published article, there was an error. A correction has been made to this sentence, which previously stated: “Chilled temperatures (below 8°C) decrease photosynthesis and leaf growth, ultimately reducing yield (Ram et al., 2007).”

The corrected sentence appears below:

“Chilled temperatures reduce photosynthesis and leaf growth, leading to lower sugarcane yields. Additionally, an 8° slope on cane farms can further affect yield (Ram et al., 2007).”

The authors apologize for this error and state that this does not change the scientific conclusions of the article in any way. The original article has been updated.

